# Life‐years lost associated with mental disorders in people with HIV: a cohort study in South Africa, Canada and the United States

**DOI:** 10.1002/jia2.70023

**Published:** 2025-08-18

**Authors:** Yann Ruffieux, John A. Joska, Raynell Lang, Chunyan Zheng, Naomi Folb, Gregory D. Kirk, Angela M. Parcesepe, Michael J. Silverberg, Sonia Napravnik, Kelly Gebo, Joseph J. Eron Jr, Brenna C. Hogan, Keri N. Althoff, Mpho Tlali, David J. Grelotti, Mona Loutfy, Peter F. Rebeiro, Mary‐Ann Davies, Matthias Egger, Gary Maartens, Andreas D. Haas

**Affiliations:** ^1^ Institute of Social and Preventive Medicine University of Bern Bern Switzerland; ^2^ HIV Mental Health Research Unit, Division of Neuropsychiatry, Department of Psychiatry and Mental Health Neuroscience Institute, University of Cape Town Cape Town South Africa; ^3^ Department of Medicine University of Calgary Calgary Alberta Canada; ^4^ Department of Epidemiology Johns Hopkins Bloomberg School of Public Health Baltimore Maryland USA; ^5^ Medscheme Cape Town South Africa; ^6^ Division of Clinical Pharmacology, Department of Medicine University of Cape Town Cape Town South Africa; ^7^ Gillings School of Global Public Health University of North Carolina at Chapel Hill Chapel Hill North Carolina USA; ^8^ Division of Research Kaiser Permanente Northern California Pleasanton California USA; ^9^ Department of Health Systems Science Kaiser Permanente Bernard J. Tyson School of Medicine Pasadena California USA; ^10^ Department of Epidemiology and Biostatistics University of California San Francisco San Francisco California USA; ^11^ Division of Infectious Disease School of Medicine University of North Carolina at Chapel Hill Chapel Hill North Carolina USA; ^12^ Department of Health Policy and Management Johns Hopkins Bloomberg School of Public Health Baltimore Maryland USA; ^13^ Centre for Integrated Data and Epidemiological Research, School of Public Health University of Cape Town Cape Town South Africa; ^14^ Department of Psychiatry UC San Diego School of Medicine San Diego California USA; ^15^ Department of Medicine University of Toronto Toronto Ontario Canada; ^16^ Maple Leaf Medical Clinic Toronto Ontario Canada; ^17^ Division of Epidemiology Department of Medicine, Vanderbilt University Medical Center Nashville Tennessee USA; ^18^ Division of Infectious Diseases Department of Medicine, Vanderbilt University Medical Center Nashville Tennessee USA; ^19^ Department of Health and Wellness Provincial Government of the Western Cape Cape Town South Africa; ^20^ Department of Infectious Diseases and Hospital Epidemiology University Hospital Zurich, University of Zurich Zurich Switzerland; ^21^ Population Health Sciences, Bristol Medical School University of Bristol Bristol UK; ^22^ Wellcome Centre for Infectious Diseases Research in Africa, Institute of Infectious Disease and Molecular Medicine University of Cape Town Cape Town South Africa

**Keywords:** Canada, mental health disorders, mortality, South Africa, substance use disorders, USA

## Abstract

**Introduction:**

People with HIV (PWH) have a high burden of mental health disorders, which contribute to increased mortality due to elevated rates of physical illness, suicide or fatal accidents. Additionally, mental health disorders can adversely affect antiretroviral therapy (ART) adherence, leading to increased HIV‐related mortality. This study aims to quantify the difference in mortality between PWH who have a mental health disorder and PWH without mental health disorders in South Africa (SA) and North America (NA).

**Methods:**

This cohort study includes PWH aged 18 years or older who initiated ART between 2000 and 2021 at a national private‐sector HIV programme in SA and 13 programmes in the United States and Canada. Mental health disorders were diagnosed according to ICD‐10 codes F10‐F99, which include psychotic disorders, bipolar disorders, depression, anxiety and substance use disorders. We estimated life‐years lost (LYL) associated with mental health disorders, quantifying the average difference in remaining life expectancy between individuals diagnosed with a mental health disorder and those without such diagnoses.

**Results:**

The study included 119,785 participants from SA (57.4% female, median age 39 years) and 142,044 from NA (85.0% male, median age 43 years). In SA, 57,999 (48.4%) were diagnosed with a mental health disorder, compared with 93,518 (65.8%) in NA. In SA, the LYL associated with any mental health disorder were 3.42 years (95% CI 2.42−4.28) in males and 2.95 years (0.67−5.95) in females. Corresponding figures for NA were 4.16 years (3.71−4.59) in males and 4.64 years (2.93−6.05) in females. In both regions, LYL were higher for psychotic and substance use disorders than for depression and anxiety. Losses were primarily due to natural deaths at CD4 counts ≥200 cells/µl, with considerable contributions at CD4 counts <200 cells/µl. Unnatural causes also contributed to the loss of life‐years in males from SA and males and females from NA.

**Conclusions:**

PWH affected by mental health disorders experience higher mortality, primarily from natural causes. LYL were associated with both immunosuppression and higher CD4 levels. Improved management of HIV and physical comorbidities among PWH affected by mental health disorders may enhance their prognosis.

## 1 INTRODUCTION

People with HIV (PWH) have a high prevalence of mental health disorders. In sub‐Saharan Africa, approximately 15% of PWH have depression [1], and >20% have anxiety [2]. In the United States, these figures are about 30% for depression and 20% for anxiety [3]. The burden of mental health disorders in PWH is significantly higher than in the general population [3].

Individuals with mental health disorders have higher mortality than the general population, experiencing a reduced life expectancy ranging from 1 to 15 years [4, 5, 6]. This elevated mortality is due to the increased risk of physical comorbidities, partly resulting from side effects of psychiatric medication, increased rates of suicide and accidents, and challenges related to access and engagement in healthcare among those with mental disorders [5, 7, 8, 9].

Among PWH, the impact of mental health disorders on mortality is of particular concern because both HIV [10, 11, 12] and mental illness [13, 14] can act as risk factors for physical comorbidities, such as diabetes or cardiovascular disease. The additive or multiplicative effects of co‐occurring HIV and mental disorders might elevate the risk of physical comorbidity, leading to non‐HIV‐related mortality, for example from a major adverse cardiovascular event. Additionally, mental health can impact adherence to antiretroviral therapy (ART) and engagement with HIV care [15, 16, 17, 18], leading to disease progression and increased AIDS‐related mortality [16].

While the links between mental health challenges and ART adherence are well‐established [15, 17, 18], the association between mental disorders and mortality among PWH remains under‐researched. The few existing studies present a mixed picture [16, 19, 20, 21]. For instance, a Danish study reported a tripling of mortality rates in PWH diagnosed with schizophrenia compared with their counterparts without schizophrenia, yet found no significant impact of schizophrenia on ART outcomes [19]. In contrast, findings from British Columbia, Canada, did not find an association between schizophrenia diagnosis and increased mortality among PWH [20]. In the United States, depression is associated with an increased likelihood of missed appointments, increased HIV viraemia and a doubling of mortality rates [21]. Similarly, a study from South Africa (SA) reported a three‐fold increase in mortality and worse HIV outcomes among those with mental illness [16].

In this study, we leverage comprehensive multi‐cohort data to estimate the life‐years lost (LYL) associated with a range of mental health diagnoses, including depression, anxiety, psychotic disorders, bipolar disorders and substance use disorders, within a South African private‐sector HIV management programme and several North American HIV cohorts. By including cohorts from two distinct settings, we aim to explore excess mortality associated with mental disorders across different HIV epidemics, healthcare systems and socio‐economic contexts.

## 2 METHODS

### 2.1 Study design and participants

We analysed routine data from the Aid for AIDS (AfA) programme and the North American AIDS Cohort Collaboration on Research and Design (NA‐ACCORD). The AfA programme is a private‐sector HIV disease management programme for insured PWH in SA. NA‐ACCORD collects data on PWH from over 200 sites across the United States and Canada [22]. As of 2021, the programme covered roughly 5% of PWH living in North America (NA) [23].

From the AfA programme, we included PWH aged 18 years or older, with known sex and date of birth, and covered by medical insurance schemes participating in AfA at any point in the study period of 1 January 2011−26 January 2021. We followed beneficiaries from the start of their insurance plan, ART initiation, their 18th birthday or 1 January 2011 (whichever came last) until the end of their insurance plan, their 85th birthday, death or 26 January 2021 (whichever came first).

From NA‐ACCORD, we included PWH aged 18 or above, with known sex and date of birth, and in care at one of 13 clinical cohorts. The study period varied by NA‐ACCORD cohort, with opening and closing dates chosen to reduce the risk of incomplete ascertainment of mental disorders [24]. Opening dates ranged from 1 January 2000 to 1 January 2014 and closing dates from 1 June 2001 to 31 December 2020. Follow‐up began at an individual's entry into the clinical cohort, ART initiation, their 18th birthday or the start of the cohort's study period (whichever came last), and stopped 18 months after their last clinical encounter in the cohort, their 85th birthday, death or the end of the cohort's study period (whichever came first). In analyses involving causes of death, we restricted the NA‐ACCORD population to the nine cohorts and the time periods where data on cause of death were available.

### 2.2 Exposures

We extracted International Classification of Diseases, 10th Revision (ICD‐10) diagnoses of mental disorders from AfA reimbursement claims from outpatient or hospital settings. We considered any mental disorders (including substance use disorder) in the range F10‐F99, excluding tobacco use disorder (F17). In further analysis, we examined any mental disorders except substance use disorders (F20‐F99), common mental disorders, including depressive disorders (F32, F33, F34.1), and anxiety disorders (F40‐F49), severe mental disorders, including psychotic disorder (F20‐F29), and bipolar disorder (F31), and substance use disorder (F10‐F16, F18, F19). In NA‐ACCORD, the corresponding disorders were identified based on ICD‐9 and ICD‐10 diagnoses recorded on inpatient and outpatient records (Table S1). Exposure variables were modelled as time‐varying. Individuals were considered unexposed until their first diagnosis for a mental disorder and exposed from their mental disorder diagnosis until the end of their follow‐up period. We carried forward any prevalent diagnosis to the start of follow‐up. Individuals could potentially contribute to multiple exposures simultaneously.

### 2.3 Outcomes

We ascertained the vital status of AfA beneficiaries based on mortality records from the National Population Register. Information on causes of death in the NA‐ACCORD was extracted from death certificates, medical records, the US National Death Index (NDI+) database and the Provincial Death Index (Canada). We distinguished between unnatural deaths, natural deaths and deaths from unknown cause using ICD codes and text searches. Unnatural causes of death included fatalities from external factors, while natural deaths were due to disease or old age, and unknown causes included deaths still under investigation or unidentified causes. In the NA‐ACCORD, we categorized unnatural deaths further into deaths from suicide (ICD‐10 codes X66‐84, Y87.0, U03), homicide (X85‐Y09, Y87.1, U01‐02) and injuries or accidents (S00‐99, T00‐T79, V00‐99, W00‐99, X00‐59, Y10‐36, Y85‐89). Natural deaths were further classified based on the latest CD4 count within the year before death, occurring at CD4 levels of <200 copies/µl, 200–349 copies/µl, >350 copies/µl or at an unknown count before death, with the assumption that deaths at low CD4 cell count were AIDS‐related.

### 2.4 Statistical analysis

We assessed the distribution of sex, baseline age, baseline calendar year and baseline CD4 cell count by region, for PWH with and without a mental health diagnosis. Baseline was defined as the start of participants’ follow‐up. We evaluated the prevalence of each type of mental health disorders by region and by sex.

We calculated LYL associated with mental health diagnoses before the age of 85 for all mental disorder groups defined above. LYL associated with a mental health disorder measure the average difference in the remaining lifetime for someone diagnosed with that disorder (exposed) compared with someone of the same age, sex and region without a mental health diagnosis (unexposed) [25, 26, 27, 28]. We used Kaplan‐Meier survival estimates to derive the remaining lifetime in people with and without a mental disorder. The cutoff of 85 years was selected due to limited follow‐up in exposed individuals beyond this age and to being above the life expectancy in both regions. When computing LYL for the NA region, we stratified the underlying survival estimates by clinical cohort. When computing LYL for the SA region, we stratified the survival estimates by calendar period (2011−2013, 2014−2021) as mortality rates were higher before 2013 and stable thereafter. We computed all‐cause LYL, and LYL disaggregated by cause of death [29]. We estimated LYL separately for each sex and region. We computed 95% confidence intervals (CI) for LYL estimates using non‐parametric bootstrap sampling with 500 iterations. We performed all statistical analyses with R 4.2.3 (R Foundation for Statistical Computing, Vienna, Austria).

In a first sensitivity analysis, we restricted the study period for the NA cohorts to that of the SA region (1 January 2011−26 January 2021). In a second sensitivity analysis, we closed the study period in both regions at the beginning of the COVID‐19 pandemic (1 March 2020).

### 2.5 Ethical considerations

Data were obtained from the International epidemiology Databases to Evaluate AIDS collaboration (IeDEA). Participating cohorts received ethical approval from their local institutional review board to contribute data to IeDEA. The Human Research Ethics Committee (HREC) of the University of Cape Town, South Africa and the Cantonal Ethics Committee Bern, Switzerland, authorized the analysis of the database. AfA members provided consent for their data to be used in research. For NA‐ACCORD, as the data are de‐identified (except for dates), a waiver of consent was obtained.

## 3 RESULTS

This study included 119,785 PWH from SA and 142,044 from NA. The characteristics of participants with and without a mental health diagnosis are displayed in Table 1. The proportion of females was higher in the SA region than in the NA region. The median age at baseline was lower in SA than in NA. The median follow‐up time was 4.3 years (IQR 2.0−5.3) in SA and 5.3 years (IQR 2.4−10.5) in NA. In South Africans of both sexes, the baseline CD4 cell count was slightly higher among individuals who received a mental health diagnosis during follow‐up than those who did not (standardized differences 0.10 and 0.02, Table S2).

**Table 1:** Characteristics of participants who did and did not receive a mental health diagnosis during follow‐up, stratified by region

	South Africa			North America		
	Received mental health diagnoses	Without mental health diagnoses	Total	Received mental health diagnoses	Without mental health diagnoses	Total
	*N* = 57,999	*N* = 61,786	*N* = 119,785	*N* = 93,518	*N* = 48,526	*N* = 142,044
Sex						
Male	22,928 (39.5%)	28,098 (45.5%)	51,026 (42.6%)	80,191 (85.7%)	40,513 (83.5%)	120,704 (85.0%)
Female	35,071 (60.5%)	33,688 (54.5%)	68,759 (57.4%)	13,327 (14.3%)	8013 (16.5%)	21,340 (15.0%)
Age at baseline^a^, years						
18–29	6151 (10.6%)	8301 (13.4%)	14,452 (12.1%)	11,381 (12.2%)	8163 (16.8%)	19,544 (13.8%)
30–39	24,459 (42.2%)	24,012 (38.9%)	48,471 (40.5%)	23,831 (25.5%)	13,186 (27.2%)	37,017 (26.1%)
40–49	18,929 (32.6%)	19,039 (30.8%)	37,968 (31.7%)	31,856 (34.1%)	14,085 (29.0%)	45,941 (32.3%)
50–59	7610 (13.1%)	8928 (14.4%)	16,538 (13.8%)	19,985 (21.4%)	8763 (18.1%)	28,748 (20.2%)
60–69	774 (1.3%)	1344 (2.2%)	2118 (1.8%)	5571 (6.0%)	3438 (7.1%)	9009 (6.3%)
70–84	76 (0.1%)	162 (0.3%)	238 (0.2%)	894 (1.0%)	891 (1.8%)	1785 (1.3%)
Median [IQR]	39.4 [33.9, 46.3]	39.4 [33.4, 46.8]	39.4 [33.7, 46.6]	43.5 [35.6, 51.0]	42.0 [33.3, 50.8]	43.1 [34.9, 51.0]
CD4 at baseline^a^, cells/µl						
<100	5244 (9.0%)	6516 (10.5%)	11,760 (9.8%)	12,152 (13.0%)	6945 (14.3%)	19,097 (13.4%)
100–199	5571 (9.6%)	6495 (10.5%)	12,066 (10.1%)	10,419 (11.1%)	5100 (10.5%)	15,519 (10.9%)
200–349	10,901 (18.8%)	11,513 (18.6%)	22,414 (18.7%)	18,881 (20.2%)	9105 (18.8%)	27,986 (19.7%)
350–499	8840 (15.2%)	8872 (14.4%)	17,712 (14.8%)	15,702 (16.8%)	7911 (16.3%)	23,613 (16.6%)
≥500	15,540 (26.8%)	14,910 (24.1%)	30,450 (25.4%)	25,436 (27.2%)	13,643 (28.1%)	39,079 (27.5%)
Unknown^b^	11,903 (20.5%)	13,480 (21.8%)	25,383 (21.2%)	10,928 (11.7%)	5822 (12.0%)	16,750 (11.8%)
Median [IQR]	369 [210, 585]	345 [187, 561]	356 [198, 574]	348 [182, 558]	353 [174, 571]	350 [180, 563]
Calendar year at baseline^a^						
2000–2003	0 (0.0%)	0 (0.0%)	0 (0.0%)	33,809 (36.2)	12,781 (26.3)	46,590 (32.8)
2004–2007	0 (0.0%)	0 (0.0%)	0 (0.0%)	15,271 (16.3)	8085 (16.7)	23,356 (16.4)
2008–2010	0 (0.0%)	0 (0.0%)	0 (0.0%)	13,532 (14.5)	6533 (13.5)	20,065 (14.1)
2011–2013	16,539 (28.5%)	23,286 (37.7%)	39,825 (33.2%)	14,261 (15.2)	8393 (17.3)	22,654 (15.9)
2014–2016	27,943 (48.2%)	21,071 (34.1%)	49,014 (40.9%)	9710 (10.4%)	6810 (14.0%)	16,520 (11.6%)
2017–2019	11,876 (20.5%)	14,697 (23.8%)	26,573 (22.2%)	6514 (7.0%)	5185 (10.7%)	11,699 (8.2%)
2020–2022	1641 (2.8%)	2732 (4.4%)	4373 (3.7%)	421 (0.5%)	739 (1.5%)	1160 (0.8%)
Died						
All‐cause mortality	3201 (5.5%)	4508 (7.3%)	7709 (6.4%)	18,171 (19.4%)	5497 (11.3%)	23,668 (16.7%)
Natural causes^c^	2818 (88.0%)	4065 (90.2%)	6883 (89.3%)	9961 (54.8%)	2983 (54.3%)	12,944 (54.7%)
CD4<200 cells/µl^d^	1319 (46.8%)	2174 (53.5%)	3493 (50.7%)	4736 (47.5%)	1510 (50.6%)	6246 (48.3%)
CD4 200–349 cells/µl^d^	409 (14.5%)	545 (13.4%)	954 (13.9%)	1771 (17.8%)	470 (15.8%)	2241 (17.3%)
CD4≥350 cells/µl^d^	803 (28.5%)	824 (20.3%)	1627 (23.6%)	2650 (26.6%)	677 (22.7%)	3327 (25.7%)
CD4 unknowns^d^	287 (10.2%)	522 (12.8%)	809 (11.8%)	804 (8.1%)	326 (10.9%)	1130 (8.7%)
Unnatural causes^c^	351 (11.0%)	394 (8.7%)	745 (9.7%)	1123 (6.2%)	198 (3.6%)	1321 (5.6%)
Unknown cause^c^	32 (1.0%)	49 (1.1%)	81 (1.1%)	7087 (39.0%)	2316 (42.1%)	9403 (39.7%)

Abbreviation: IQR, interquartile range.

^a^ Baseline is defined as the start of a person's follow‐up time.

^b^ No CD4 cell count measurement available between 180 days before and 30 days after the individual's baseline.

^c^ Denominators for percentages are total deaths in that group.

^d^ Denominators for percentages are total natural deaths in that group.

Table 2 shows the prevalence of mental health disorders by sex and region. A total of 57,999 (48.4%) PWH in SA received a mental health diagnosis, with a higher proportion in females (51.0%) than in males (44.9%). In NA, 93,518 (65.1%) PWH received a mental health diagnosis, including 66.4% of males and 62.5% of females. Depressive and anxiety disorders were the most common diagnoses in both regions. Substance use disorder diagnoses were prevalent in NA (37.2%), but uncommon in SA (1.5%). Psychiatric comorbidity was common in both regions: among individuals with a diagnosis of a mental disorder, 44.4% in SA and 65.3% in NA received a diagnosis of another mental health disorder during follow‐up (Figure S1). The characteristics of the 77,582 individuals from the North American population with available cause of death data are shown in Tables S3 and S4.

**Table 2:** Number and proportion of participants who received a diagnosis for mental health disorder of a given type during follow‐up, by region and sex

	South Africa			North America		
Diagnosis	Male (*N* = 51,026)	Female (*N* = 68,759)	Total (*N* = 119,786)	Male (*N* = 120,704)	Female (*N* = 21,340)	Total (*N* = 142,044)
Any mental disorder	22,928 (44.9%)	35,071 (51.0%)	57,999 (48.4%)	80,191 (66.4%)	13,327 (62.5%)	93,518 (65.8%)
Severe mental disorders	1468 (2.9%)	2382 (3.5%)	3850 (3.2%)	19,279 (16.0%)	3399 (15.9%)	22,678 (16.0%)
Psychotic disorders	505 (1.0%)	651 (0.9%)	1156 (1.0%)	12,742 (10.6%)	1845 (8.6%)	14,587 (10.3%)
Bipolar disorder	1093 (2.1%)	1919 (2.8%)	3012 (2.5%)	10,850 (9.0%)	2250 (10.5%)	13,100 (9.2%)
Common mental disorders	20,890 (40.9%)	33,457 (48.7%)	54,347 (45.4%)	67,587 (56.0%)	11,382 (53.3%)	78,969 (55.6%)
Depressive disorders	12,254 (24.0%)	20,717 (30.1%)	32,971 (27.5%)	56,643 (46.9%)	9852 (46.2%)	66,495 (46.8%)
Anxiety disorders	15,679 (30.7%)	25,940 (37.7%)	41,619 (34.7%)	47,112 (39.0%)	6616 (31.0%)	53,728 (37.8%)
Substance use disorders	1185 (2.3%)	614 (0.9%)	1799 (1.5%)	46,427 (38.5%)	6383 (29.9%)	52,810 (37.2%)
Any mental disorder except substance use disorder	22,780 (44.6%)	35,026 (50.9%)	57,806 (48.3%)	70,687 (58.6%)	12,094 (56.7%)	82,781 (58.3%)

*Note*: Individuals can contribute to multiple categories of mental health disorders.

**Figure 1:**
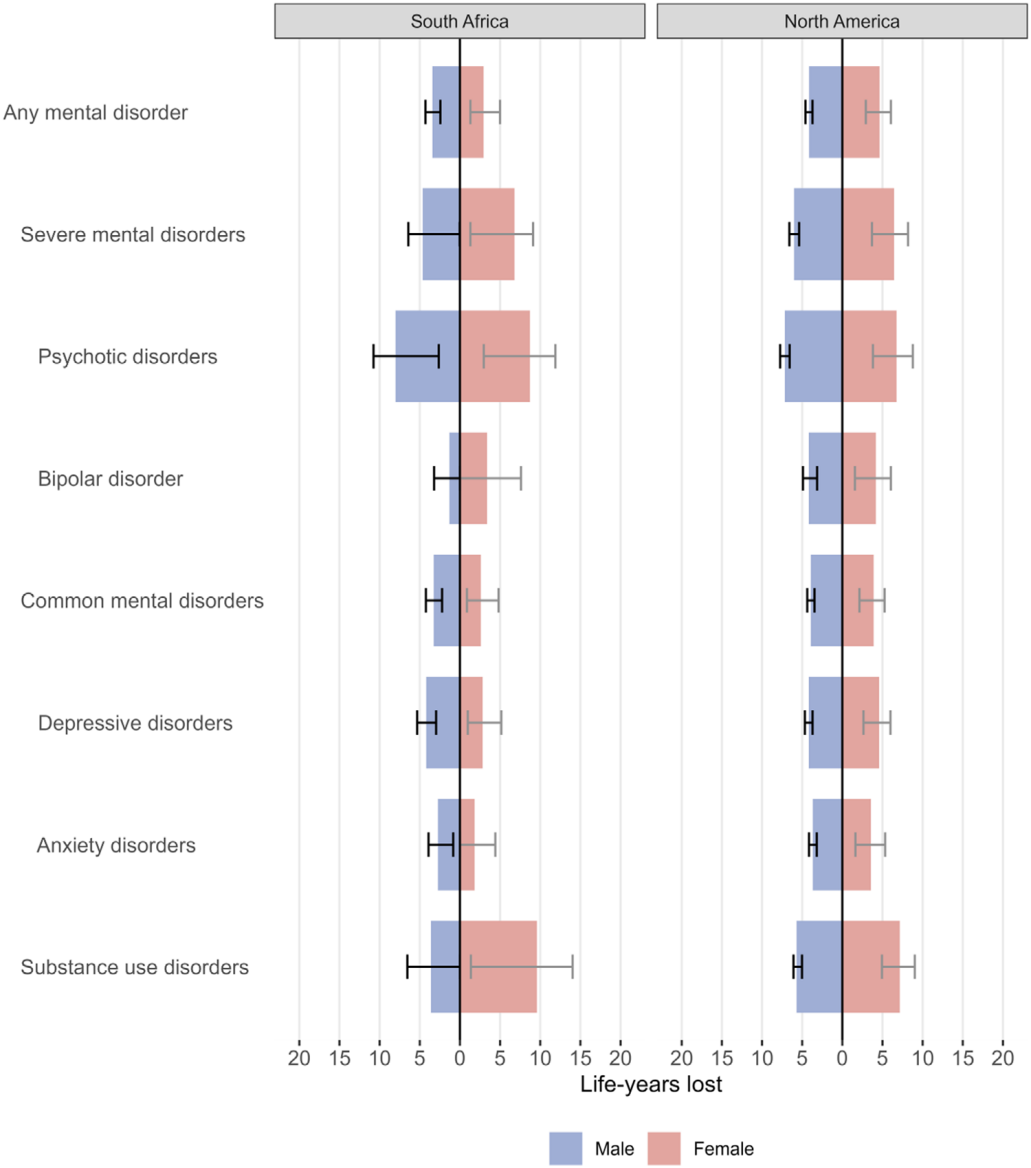
Life‐years lost associated with mental health diagnoses. *Note*: Data are stratified by region and sex. The error bars represent 95% confidence intervals. Confidence limits with negative values are truncated at 0 in the figure.

Figure 1 shows the LYL associated with a mental health diagnosis in PWH by region and sex. Compared with individuals without a mental health diagnosis of the same age, sex and region, the remaining lifespan after the mental health diagnosis was 3.42 years (95% CI 2.42−4.28) shorter in South African males, 2.95 years (95% CI 1.30−4.99) shorter in South African females, 4.16 years (95% CI 3.71−4.59) shorter in North American males and 4.64 years (95% CI 2.93−6.05) shorter in North American females (Table S5). Psychotic disorders were associated with 7−9 LYL, while substance use disorders were associated with 4−10 LYL. In South African males and in North American of both sexes, PWH diagnosed with a depressive disorder lost more than four life‐years compared with PWH with no diagnosis of a mental disorder. Bipolar and anxiety disorders were associated with fewer than four LYL in SA and fewer than five LYL in NA. There were no major changes in all‐cause LYL when restricting the study period for the NA region to 1 January 2011−26 January 2021 (Table S6). When ending the study period before the beginning of the COVID‐19 pandemic, the LYL associated with mental disorders increased to 3.60 (95% CI 1.33−5.81) in South African females but remained similar for other groups (Table S7).

**Figure 2:**
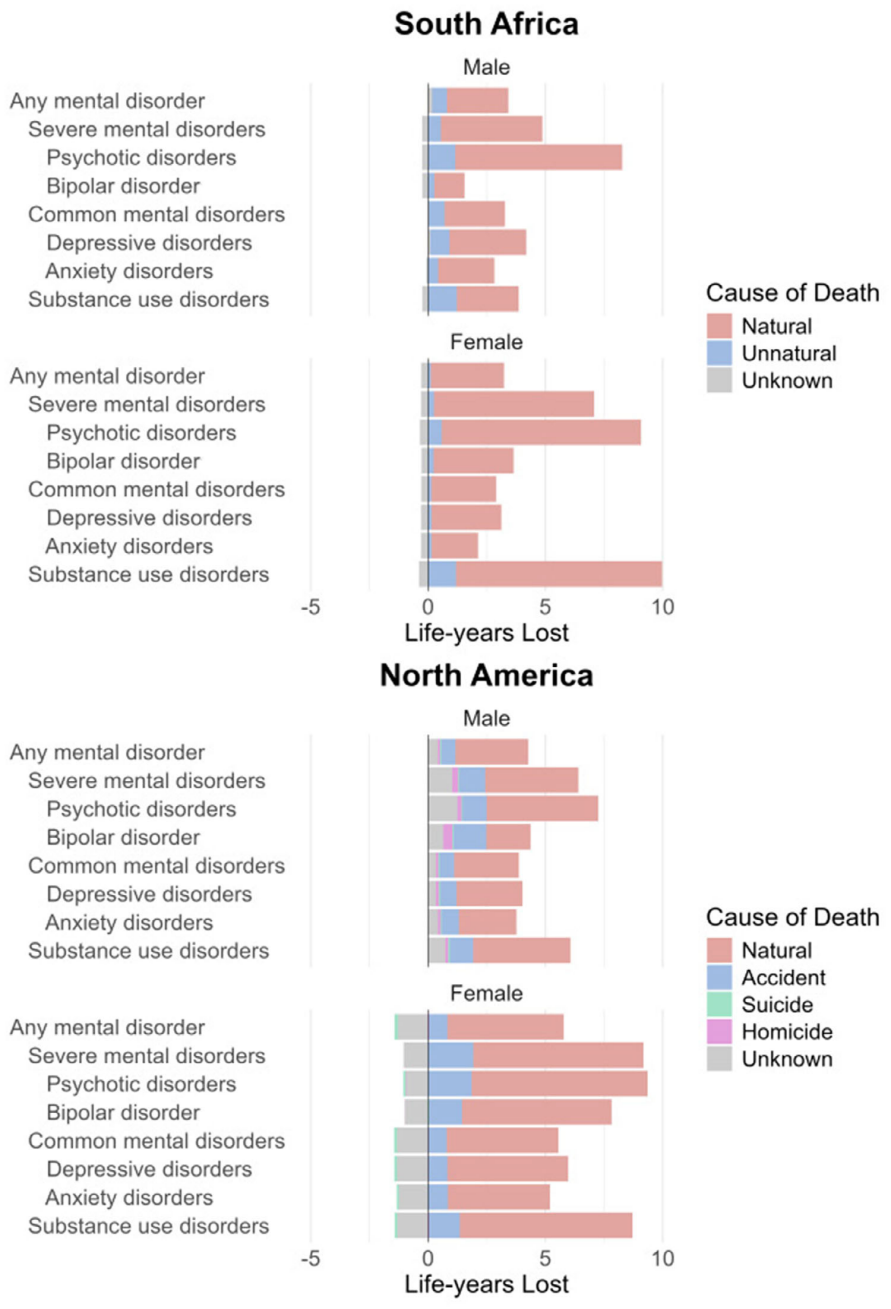
Life‐years lost (LYL) due to specific causes of death associated with mental health diagnoses. *Note*: Data are stratified by region and sex. Negative LYL signify a longer lifetime among those with mental health diagnoses compared with those without. Data from North America are restricted to the study population with available cause of death data.

Across both regions and both sexes, the majority of LYL associated with mental health disorders in PWH was due to natural causes of death (Figure 2, Tables S8 and S9). For South African males with any mental disorder, 2.62 (76.7%) LYL were due to natural causes. In North American males, the figure was 3.11 (72.7%) LYL. In South African females, unnatural causes had a minor contribution (0.12 LYL) to the all‐cause LYL associated with any mental disorder, but in North American females, accidents accounted for 0.78 LYL. In South African of both sexes, substance use disorders were associated with more than one LYL due to unnatural causes (Figure 2 and Table S8). In NA, accidental deaths contributed to over 20% of the all‐cause LYL associated with severe disorders and substance use disorder, while other unnatural causes accounted for minimal LYL (Figure 2 and Table S9).

**Figure 3:**
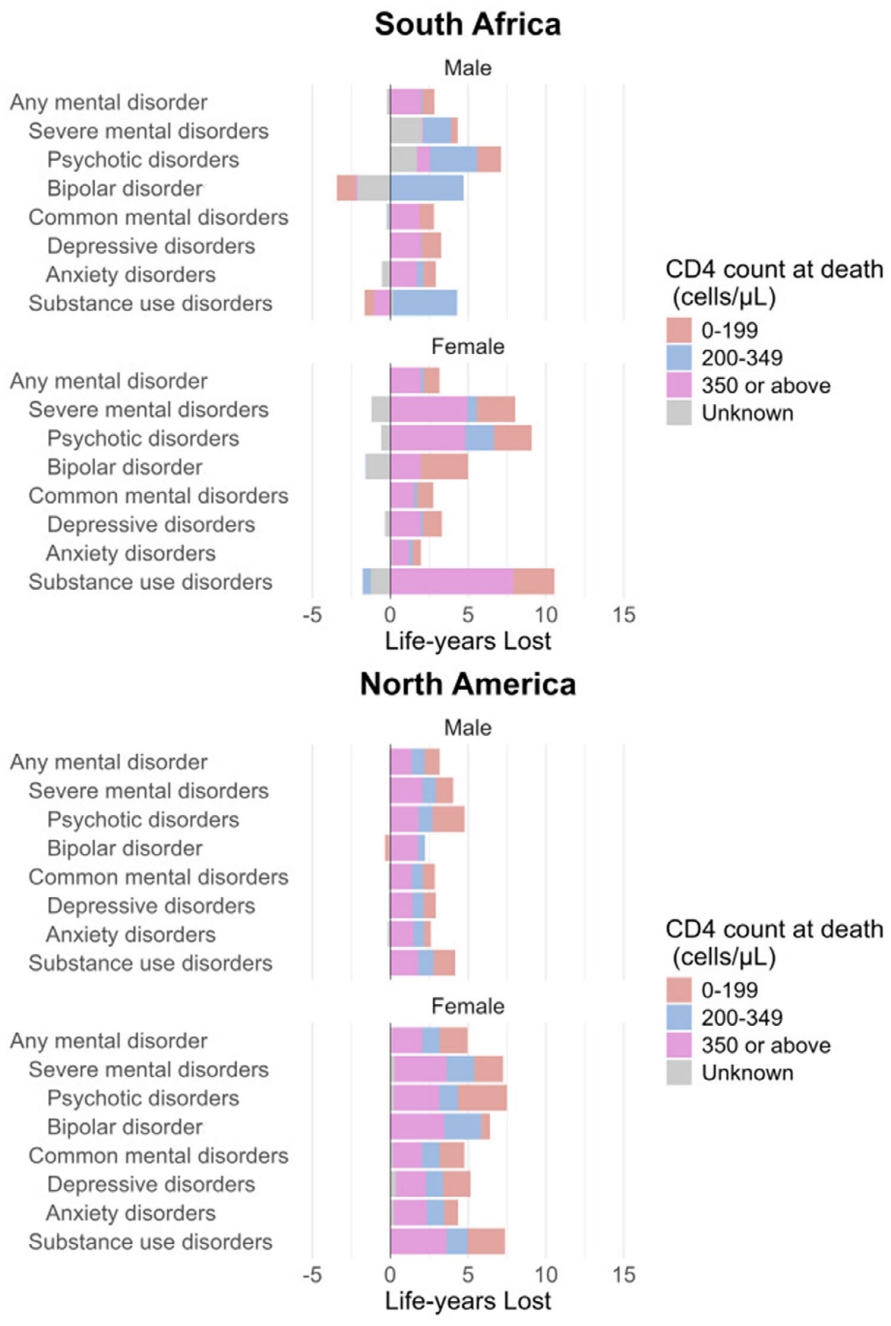
Life‐years lost (LYL) due to natural deaths associated with mental health diagnoses. *Note*: Data are stratified by sex, and the estimates are further disaggregated into four causes of natural death, based on the last CD4 cell count within a year of death. Negative LYL signify a longer lifespan among those with mental health diagnoses compared with those without. Data from North America are restricted to the study population with available cause of death data.

The median CD4 cell count at the time of a natural death was higher in PWH with a mental health diagnosis compared to PWH without such a diagnosis (Table S10). Figure 3 and Tables S11 and S12 show LYL due to natural deaths associated with mental health diagnoses, disaggregated according to CD4 cell count before death. In SA, most LYL associated with any mental disorder occurred at CD4 counts ≥350 cells/µl, whereas in NA, most losses occurred at CD4 counts <350 cells/µl.

## 4 DISCUSSION

This multi‐cohort study of over 260,000 individuals demonstrated that PWH with mental health disorders experience substantially higher mortality compared with PWH without such conditions, resulting in the loss of several life‐years. The magnitude of the LYL was significantly greater in those diagnosed with substance use or psychotic disorders, compared with those with common mental disorders such as depression and anxiety. Substantial losses in life‐years among those with mental health disorders were observed in SA and in the United States and Canada. In both regions, losses were primarily due to natural deaths occurring at CD4 counts ≥200 cells/µl, with considerable contributions at CD4 counts <200 cells/µl. Unnatural causes also contributed to the loss of life‐years in males from SA and males and females from NA.

We found a higher excess mortality associated with mental disorders in NA. This may be explained by the higher prevalence of substance use disorders, and their associated health risks (e.g. overdose, unsafe injection practices) and comorbidities, or by differences in socio‐economic status between NA cohorts of key populations and the private‐sector SA cohort of insured and employed individuals in a setting with a generalized HIV epidemic. This excess mortality risk associated with mental disorders in PWH has been noted in other studies. A study of PWH in the South African public sector found that the mortality rate in people with a history of mental disorders was three times that of people without [16]. A US‐based study found that prolonged depression was associated with increased mortality rates in PWH [21]. Of note, our estimates for LYL associated with mental disorders in PWH were similar to those reported in a comparable study of a general private‐sector population in SA, where HIV prevalence is approximately 7% [6], but were smaller than those from a study of the general population in Denmark [27].

Four potential mechanisms may explain the observed losses of life‐years among individuals with mental health disorders. First, people with mental health disorders may have initiated ART later and at lower CD4 counts than those without such conditions, leading to a poorer prognosis [30, 31]. However, our findings do not support this hypothesis, as individuals diagnosed with mental disorders had a higher baseline CD4 cell count than those who did not receive mental health diagnoses.

A second factor that could potentially explain the shorter lifespan of people with mental health disorders might be their lower ART adherence [15] or lower engagement in care [16], leading to increased HIV‐related mortality. In both regions, a considerable number of LYL occurred at CD4 counts <200 cells/µl. However, in SA, most natural deaths that led to the loss of life‐years occurred at CD4 counts ≥350 cells/µl, suggesting that these losses were not primarily driven by HIV‐related mortality. In the United States and Canada, slightly more life‐years were lost at CD4 counts <350 cells/µl, potentially indicating HIV‐related mortality [32]. A possible reason for the higher number of LYL at lower CD4 counts in NA compared to SA could be the greater prevalence of psychotic and substance use disorders. These disorders are strongly associated with losses at CD4 counts <200 cells/µl, potentially due to challenges in adherence and retention, which are particularly pronounced in this group [15, 16].

A third potential factor contributing to the loss of life‐years in individuals with mental disorders is the increased likelihood of suicide and accidental deaths in this population [5, 33]. Our findings indicate that in SA, the impact of unnatural causes on the overall losses of life‐years among females with mental disorders is negligible and among males considerable, but not a major driver, with notable exceptions among males with psychotic disorders and females with substance use disorders. In NA, while accidental deaths constitute a modest yet significant portion of the losses in life‐years, other forms of unnatural death, including suicide and homicide, do not contribute substantially to the overall mortality burden.

A fourth contributing factor to the reduced lifespan of individuals with mental health disorders is the higher incidence of physical illnesses in this group, potentially exacerbated by side effects of psychiatric medication [7], often coupled with suboptimal management of these conditions [8, 34, 35], leading to increased mortality from natural, non‐HIV‐related causes [9, 36]. This pathway is particularly significant for individuals living with HIV, as the virus itself [10, 11, 12] and the side effects of ART [12] can increase the risk for certain non‐communicable diseases such as cardiovascular diseases or diabetes. Additionally, among substance users, unsafe drug use behaviours may lead to a higher prevalence of co‐infections such as hepatitis B or C, further contributing to increased mortality due to physical illness [37].

Our findings should be interpreted considering the following limitations. First, we assessed participants’ mental health status based on ICD diagnoses from routinely collected medical or administrative records. This approach does not capture individuals with undiagnosed mental disorders who may be less severely ill than those diagnosed, potentially leading to an overestimation of the LYL associated with mental disorders. Administrative records may be subject to diagnostic or administrative errors; however, diagnoses from such data generally demonstrate acceptable positive predictive value for research diagnoses [38]. Sensitivity appears to be high, as indicated by the high prevalence estimates observed, consistent with expectations for PWH [1, 2, 3]. High sensitivity is plausible given that all individuals in our cohorts were enrolled in HIV care and regularly seen by healthcare providers who are likely to be attuned to mental health conditions. Substance use disorders (including alcohol use disorders) in the South African cohort are a notable exception. Fewer than 2% of participants were diagnosed with a substance use disorder, substantially lower than expected [39]. The observed prevalence of substance use disorders in SA likely reflects underdiagnosis, potentially leading to underestimation of LYL, given the strong association between substance use disorders and mortality. Underdiagnosis of substance use disorders in SA may also partly explain the overall lower excess LYL associated with mental disorders in SA compared to NA. However, considering that most individuals with substance use disorders have comorbid mental disorders, which are well‐ascertained in this cohort, the extent of underestimation of the overall LYL associated with mental disorders in SA is likely limited. Second, our analysis did not account for remission in mental illness, potentially leading to an underestimation of LYL. Third, our analysis of cause‐specific LYL is reliant on the quality of cause of death information. In SA, we sourced this data from the national vital registration system. A national validation study indicated that mortality from unnatural causes is slightly underestimated, accounting for 11.2% in the vital registration system, 12.6% in InterVA‐5 coded verbal autopsies and 12.9% based on doctor reviews of verbal autopsies [40]. In NA, cause of death information was obtained from multiple sources, including death certificates, medical record reviews and Provincial Death Indices. While death ascertainment may vary between cohorts, we stratified by cohort to ensure that exposed and unexposed individuals are compared under similar conditions. Data on the specific cause of death was unavailable in both regions. For natural deaths, we used the CD4 count at death as a proxy for AIDS‐related mortality. Although not all deaths at low CD4 counts are AIDS‐related, a CD4 count below 200 might be a valid proxy for AIDS‐related mortality due to extremely strong associations between low CD4 and AIDS‐related mortality [32]. Fourth, the South African data, sourced from private‐sector insurance schemes, are not representative of individuals in public sector care. Persons using the public healthcare sector typically have a lower socio‐economic status and may be at a higher risk of experiencing poor mental health and HIV treatment outcomes compared to those who are accessing private healthcare services. Lastly, the LYL metric, useful for identifying individuals at increased risk of mortality who require specific clinical attention, is descriptive and does not imply causality. The estimates do not isolate the influence of individual mental disorders but also include the impact of co‐occurring exposures, such as comorbid mental conditions. Additionally, LYL estimates may be influenced by confounding, and could reflect the effects of common causes of mental disorders and mortality, such as lower socio‐economic status.

## 5 CONCLUSIONS

In conclusion, our study demonstrates that individuals with HIV and co‐occurring mental health disorders experience considerable losses of life‐years compared to those with HIV but without a mental disorder. These losses are multifactorial and vary in size depending on the mental disorder. For people with common mental disorders, most losses are due to natural deaths at CD4 counts of above 200. In contrast, among individuals with severe mental disorders and substance use disorders, unnatural deaths and natural deaths at CD4 cell counts below 200 play a more significant role. To narrow the mortality gap for people with mental health disorders, interventions should address the specific underlying causes. For individuals with common mental disorders, interventions should focus on improved management of physical comorbidities to reduce mortality at higher CD4 cell counts, which are unlikely to be HIV‐related. For example, cardiovascular disease is a major contributor to excess mortality in people with depression [41]. Initiatives such as the REPRIEVE trial, which evaluates the use of statin therapy to prevent cardiovascular events among PWH, offer avenues to reduce the resulting mortality gap [42]. While patients should always be assessed individually without implicit bias regarding their medication adherence, there should be a greater emphasis on enhancing HIV care for patient groups with considerable losses at low CD4 cell counts, such as those with severe mental disorders or substance use issues, to reduce HIV‐related mortality.

## COMPETING INTERESTS

The authors report no competing interests.

## AUTHORS’ CONTRIBUTIONS

KNA, M‐AD, ME and ADH obtained funding for the study. YR and ADH wrote the first draft of the study protocol, which was subsequently reviewed and revised by all authors. YR performed the statistical analyses. YR and ADH wrote the first draft of the manuscript, with significant input from all authors. All authors contributed to the interpretation of the results and critically revised the manuscript for intellectual content. All authors have approved the final version of the manuscript.

## FUNDING

IeDEA Southern Africa: The research reported in this publication was supported by the U.S. National Institutes of Health's National Institute of Allergy and Infectious Diseases, the Eunice Kennedy Shriver National Institute of Child Health and Human Development, the National Cancer Institute, the National Institute of Mental Health, the National Institute on Drug Abuse, the National Heart, Lung, and Blood Institute, the National Institute on Alcohol Abuse and Alcoholism, the National Institute of Diabetes and Digestive and Kidney Diseases, and the Fogarty International Center [U01AI069924 to ME and M‐AD], and the Swiss National Science Foundation [193381 to ADH and 189498 to ME].

NA‐ACCORD: This work was supported by National Institutes of Health grants U01AI069918, F31AI124794, F31DA037788, G12MD007583, K01AI093197, K01AI131895, K23EY013707, K24AI065298, K24AI118591, K24DA000432, KL2TR000421, N01CP01004, N02CP055504, N02CP91027, P30AI027757, P30AI027763, P30AI027767, P30AI036219, P30AI050409, P30AI050410, P30AI094189, P30AI110527, P30MH62246, R01AA016893, R01DA011602, R01DA012568, R01 AG053100, R24AI067039, U01AA013566, U01AA020790, U01AI038855, U01AI038858, U01AI068634, U01AI068636, U01AI069432, U01AI069434, U01DA03629, U01DA036935, U10EY008057, U10EY008052, U10EY008067, U01HL146192, U01HL146193, U01HL146194, U01HL146201, U01HL146202, U01HL146203, U01HL146204, U01HL146205, U01HL146208, U01HL146240, U01HL146241, U01HL146242, U01HL146245, U01HL146333, U24AA020794,U54MD007587, UL1RR024131, UL1TR000004, UL1TR000083, Z01CP010214 and Z01CP010176; contracts CDC‐200‐2006‐18797 and CDC‐200‐2015‐63931 from the Centers for Disease Control and Prevention, USA; contract 90047713 from the Agency for Healthcare Research and Quality, USA; contract 90051652 from the Health Resources and Services Administration, USA; grants CBR‐86906, CBR‐94036, HCP‐97105 and TGF‐96118 from the Canadian Institutes of Health Research, Canada; Ontario Ministry of Health and Long Term Care; and the Government of Alberta, Canada. Additional support was provided by the National Institute Of Allergy And Infectious Diseases (NIAID), National Cancer Institute (NCI), National Heart, Lung, and Blood Institute (NHLBI), Eunice Kennedy Shriver National Institute Of Child Health & Human Development (NICHD), National Human Genome Research Institute (NHGRI), National Institute for Mental Health (NIMH) and National Institute on Drug Abuse (NIDA), National Institute On Aging (NIA), National Institute Of Dental & Craniofacial Research (NIDCR), National Institute Of Neurological Disorders And Stroke (NINDS), National Institute Of Nursing Research (NINR), National Institute on Alcohol Abuse and Alcoholism (NIAAA), National Institute on Deafness and Other Communication Disorders (NIDCD), and National Institute of Diabetes and Digestive and Kidney Diseases (NIDDK).

## DISCLAIMER

The content is solely the authors’ responsibility and does not necessarily represent the official views of the funders.

## Supporting information




**Table S1**: Classification of mental health diagnoses.
**Table S2**: Baseline CD4 cell count by region, sex and mental health status.
**Table S3**: Characteristics of participants from North America who did and did not receive a mental health diagnosis during follow‐up, restricted to the study population with available cause of death data.
**Table S4**: Number and proportion of participants from North America who received mental health diagnoses of a given type during follow‐up, by sex, restricted to the study population with available cause of death data.
**Table S5**: Life‐years lost (LYL) associated with mental health diagnoses. Data are stratified by region and sex.
**Table S6**: All‐cause life‐years lost (LYL) associated with mental health diagnoses in the North America region between Jan 1, 2011 and Jan 26, 2021.
**Table S7**: All‐cause life‐years lost (LYL) associated with mental health diagnoses before the COVID‐19 pandemic (March 1, 2020).
**Table S8**: Life‐years lost (LYL) associated with mental health diagnoses by cause of death in South Africa.
**Table S9**: Life‐years lost (LYL) associated with mental health diagnoses by cause of death and sex in North America.
**Table S10**: CD4 cell count at the time of a death from natural causes, by region, sex and mental health status.
**Table S11**: Life‐years lost (LYL) due to natural causes associated with mental health diagnoses in South Africa, by sex and by CD4 cell count at death.
**Table S12**: Life‐years lost (LYL) due to natural causes associated with mental health diagnoses in North America, by sex and by CD4 cell count at death.
**Figure S1**: Psychiatric comorbidity among participants from South Africa and North America.

## Data Availability

Data were obtained from the International epidemiology Databases to Evaluate AIDS collaboration. Due to legal and ethical restrictions, the data cannot be made publicly available. For inquiries about the data, readers can contact IeDEA Southern Africa through the online form available at https://www.iedea‐sa.org/contact‐us/.
