# Supplementary material for: Life‐years lost associated with mental disorders in people with HIV: a cohort study in South Africa, Canada and the United States
Source: J Int AIDS Soc. 2025 Aug 18;28(8):e70023. doi: 10.1002/jia2.70023 (PMC12361348; doi:10.1002/jia2.70023)
Supplement: Supplementary file 1 — Table S1: Classification of mental health diagnoses. Table S2: Baseline CD4 cell count by region, sex and mental health status. Table S3: Characteristics of participants from North America who did and did not receive a mental health diagnosis during follow‐up, restricted to the study population with available cause of death data. Table S4: Number and proportion of participants from North America who received mental health diagnoses of a given type during follow‐up, by sex, restricted to the study population with available cause of death data. Table S5: Life‐years lost (LYL) associated with mental health diagnoses. Data are stratified by region and sex. Table S6: All‐cause life‐years lost (LYL) associated with mental health diagnoses in the North America region between Jan 1, 2011 and Jan 26, 2021. Table S7: All‐cause life‐years lost (LYL) associated with mental health diagnoses before the COVID‐19 pandemic (March 1, 2020). Table S8: Life‐years lost (LYL) associated with mental health diagnoses by cause of death in South Africa. Table S9: Life‐years lost (LYL) associated with mental health diagnoses by cause of death and sex in North America. Table S10: CD4 cell count at the time of a death from natural causes, by region, sex and mental health status. Table S11: Life‐years lost (LYL) due to natural causes associated with mental health diagnoses in South Africa, by sex and by CD4 cell count at death. Table S12: Life‐years lost (LYL) due to natural causes associated with mental health diagnoses in North America, by sex and by CD4 cell count at death. Figure S1: Psychiatric comorbidity among participants from South Africa and North America. [file JIA2-28-e70023-s001.docx]

Life-Years Lost Associated with Mental Health Disorders in People with HIV: A Cohort Study in South Africa, Canada, and the United States

Supplemental Appendix

**Supplemental Table 1**: Classification of mental health diagnoses.

| **#** | **Label** | **Definition** | **ICD-10 codes** |
| --- | --- | --- | --- |
| 1 | Substance use disorders | Substance abuse of or dependence to any psychoactive substance except tobacco | F10-F16, F18-F19 |
| 2 | Psychotic disorders | Schizophrenia, schizotypal and delusional disorders | F20-F29 |
| 3 | Bipolar disorder | Bipolar affective disorder | F31 |
| 4 | Depressive disorders | Depression and dysthymia | F32, F33, F34.1 |
| 5 | Anxiety disorders | Neurotic, stress-related and somatoform disorders | F40-F48 |
| 6 | Other mental disorders | Other non-organic mental disorders not classified elsewhere | F50-F99 |
|  | Severe mental disorders | Psychotic disorders [2] and bipolar disorder [3] |  |
|  | Common mental disorders | Depressive disorders [4] and anxiety disorders [5] |  |
|  | Any mental disorder except substance use disorders | Any non-organic mental health including psychotic disorders [2], bipolar disorder [3], depressive disorders [4], anxiety disorders [5], and other mental disorders [6] |  |
|  | Any mental disorder | Any non-organic mental health or substance use disorder including substance use disorders [1], psychotic disorders [2], bipolar disorder [3], depressive disorders [4], anxiety disorders [5], and other mental disorders [6] |  |
|  | No mental disorder | No non-organic mental health or substance use disorder |  |

**Supplemental Table 2**: Baseline CD4 cell count by region, sex, and mental health status.

|  | **Male** | |  | **Female** | |
| --- | --- | --- | --- | --- | --- |
|  | Received mental health diagnoses | Without mental health diagnoses |  | Received mental health diagnoses | Without mental health diagnoses |
| **South Africa** | N=22928 | N=28098 |  | N=35071 | N=33688 |
| Baseline CD4, cells/µL^a^ |  |  |  |  |  |
| <100 | 2770 (12.1%) | 3865 (13.8%) |  | 2474 (7.1%) | 2651 (7.9%) |
| 100-199 | 2601 (11.3%) | 3533 (12.6%) |  | 2970 (8.5%) | 2962 (8.8%) |
| 200-349 | 4499 (19.6%) | 5558 (19.8%) |  | 6402 (18.3%) | 5955 (17.7%) |
| 350-499 | 3402 (14.8%) | 3881 (13.8%) |  | 5438 (15.5%) | 4991 (14.8%) |
| ≥500 | 4762 (20.8%) | 4897 (17.4%) |  | 10778 (30.7%) | 10013 (29.7%) |
| Unknown^b^ | 4894 (21.3%) | 6364 (22.6%) |  | 7009 (20.0%) | 7116 (21.1%) |
| Median [IQR] | 321 [167, 514] | 293 [145, 476] |  | 407 [243, 631] | 399 [229, 626] |
| Standardized difference^c^ | 0.102 |  |  | 0.022 |  |
| **North America** | N=80191 | N=40513 |  | N=13327 | N=8013 |
| Baseline CD4, cells/µL^a^ |  |  |  |  |  |
| <100 | 10423 (13.0%) | 5805 (14.3%) |  | 1729 (13.0%) | 1140 (14.2%) |
| 100-199 | 8941 (11.1%) | 4273 (10.5%) |  | 1478 (11.1%) | 827 (10.3%) |
| 200-349 | 16172 (20.2%) | 7641 (18.9%) |  | 2709 (20.3%) | 1464 (18.3%) |
| 350-499 | 13533 (16.9%) | 6629 (16.4%) |  | 2169 (16.3%) | 1282 (16.0%) |
| ≥500 | 21607 (26.9%) | 11204 (27.7%) |  | 3829 (28.7%) | 2439 (30.4%) |
| Unknown^b^ | 9515 (11.9%) | 4961 (12.2%) |  | 1413 (10.6%) | 861 (10.7%) |
| Median [IQR] | 348 [182, 555] | 351 [173, 566] |  | 352 [186, 580] | 363 [180, 592] |
| Standardized difference^c^ | 0.000 |  |  | -0.004 |  |

Abbreviations; IQR, interquartile range. ^a^ Baseline is defined as the start of an individual's follow-up period. ^b^ No CD4 cell count measurement available between 180 days before and 30 days after the start of the individual’s baseline. ^c^ Comparing baseline CD4 between people who received a mental health diagnosis and people who didn’t.

**Supplemental Table 3**: Characteristics of participants from North America who did and did not receive a mental health diagnosis during follow-up, restricted to the study population with available cause of death data.

|  | **Received mental health diagnoses** | **Without mental health diagnoses** | **Total** |
| --- | --- | --- | --- |
|  | N=54648 | N=22934 | N=77582 |
| Sex |  |  |  |
| Male | 49607 (90.8%) | 20844 (90.9%) | 70451 (90.8%) |
| Female | 5041 (9.2%) | 2090 (9.1%) | 7131 (9.2%) |
| Age at baseline^a^, years |  |  |  |
| 18-29 | 5092 (9.3%) | 2902 (12.7%) | 7994 (10.3%) |
| 30-39 | 12383 (22.7%) | 5880 (25.6%) | 18263 (23.5%) |
| 40-49 | 19700 (36.0%) | 6769 (29.5%) | 26469 (34.1%) |
| 50-59 | 13259 (24.3%) | 4730 (20.6%) | 17989 (23.2%) |
| 60-69 | 3638 (6.7%) | 2091 (9.1%) | 5729 (7.4%) |
| 70-84 | 576 (1.1%) | 562 (2.5%) | 1138 (1.5%) |
| Median [IQR] | 45.0 [37.5, 52.0] | 43.8 [35.3, 52.7] | 44.6 [36.8, 52.1] |
| CD4 at baseline^a^, cells/µL |  |  |  |
| <100 | 7025 (12.9%) | 3175 (13.8%) | 10200 (13.1%) |
| 100-199 | 6177 (11.3%) | 2338 (10.2%) | 8515 (11.0%) |
| 200-349 | 11080 (20.3%) | 4202 (18.3%) | 15282 (19.7%) |
| 350-499 | 9109 (16.7%) | 3621 (15.8%) | 12730 (16.4%) |
| ≥500 | 14371 (26.3%) | 6419 (28.0%) | 20790 (26.8%) |
| Unknown^b^ | 6886 (12.6%) | 3179 (13.9%) | 10065 (13.0%) |
| Median [IQR] | 343 [181, 551] | 355 [177, 576] | 347 [180, 558] |
| Calendar year at baseline^a^ |  |  |  |
| 2000-2003 | 24863 (45.5) | 8538 (37.2) | 33401 (43.1) |
| 2004-2007 | 8398 (15.4) | 3741 (16.3) | 12139 (15.6) |
| 2008-2010 | 8175 (15.0) | 2914 (12.7) | 11089 (14.3) |
| 2011-2013 | 6952 (12.7) | 2856 (12.5) | 9808 (12.6) |
| 2014-2016 | 4110 (7.5%) | 2543 (11.1%) | 6653 (8.6%) |
| 2017-2019 | 2041 (3.7%) | 2103 (9.2%) | 4144 (5.3%) |
| 2020-2022 | 109 (0.2%) | 239 (1.0%) | 348 (0.4%) |
| Died |  |  |  |
| All-cause mortality | 11608 (21.2%) | 3264 (14.2%) | 14872 (19.2%) |
| Natural causes^c^ | 9268 (79.8%) | 2742 (84.0%) | 12010 (80.8%) |
| CD4<200 cells/µL^d^ | 4409 (47.6%) | 1390 (50.7%) | 5799 (48.3%) |
| CD4 200-349 cells/µL^d^ | 1645 (17.7%) | 424 (15.5%) | 2069 (17.2%) |
| CD4≥350 cells/µL^d^ | 2462 (26.6%) | 626 (22.8%) | 3088 (25.7%) |
| CD4 unknown^d^ | 752 (8.1%) | 302 (11.0%) | 1054 (8.8%) |
| Unnatural causes^c^ | 1338 (11.5%) | 337 (10.3%) | 1675 (11.3%) |
| Unknown causes^c^ | 1002 (8.6%) | 185 (5.7%) | 1187 (8.0%) |

Abbreviations: IQR, interquartile range ^a^ Baseline is defined as the start of a person’s follow-up time. ^b^ No CD4 cell count measurement available between 180 days before and 30 days after the individual’s baseline. ^c^ Denominators for percentages are total deaths in that group. ^d^ Denominators for percentages are total natural deaths in that group.

**Supplemental Table 4:** Number and proportion of participants from North America who received mental health diagnoses of a given type during follow-up, by sex, restricted to the study population with available cause of death data.

| **Diagnosis** | **Male**  **(N=70451)** | **Female**  **(N=7131)** | **Total**  **(N=77582)** |
| --- | --- | --- | --- |
| Any mental disorder | 49607 (70.4%) | 5041 (70.7%) | 54648 (70.4%) |
| Severe mental disorders | 11989 (17.0%) | 1229 (17.2%) | 13218 (17.0%) |
| Psychotic disorders | 8379 (11.9%) | 671 (9.4%) | 9050 (11.7%) |
| Bipolar disorder | 6394 (9.1%) | 853 (12.0%) | 7247 (9.3%) |
| Common mental disorders | 42941 (61.0%) | 4468 (62.7%) | 47409 (61.1%) |
| Depressive disorders | 36201 (51.4%) | 3962 (55.6%) | 40163 (51.8%) |
| Anxiety disorders | 30629 (43.5%) | 2746 (38.5%) | 33375 (43.0%) |
| Substance use disorders | 28470 (40.4%) | 2352 (33.0%) | 30822 (39.7%) |
| Any mental disorder except substance use disorders | 44360 (63.0%) | 4654 (65.3%) | 49014 (63.2%) |

**Supplemental Table 5:** Life-years lost (LYL) associated with mental health diagnoses. Data are stratified by region and sex.

|  |  | **Life-years lost ^a^ [95% confidence intervals]** | |
| --- | --- | --- | --- |
| **Diagnosis** | **Sex** | **South Africa** | **North America** |
| Any mental disorder | Male | 3.42 [2.44; 4.29] | 4.16 [3.71; 4.59] |
|  | Female | 2.95 [1.30; 4.99] | 4.64 [2.93; 6.05] |
| Severe mental disorders | Male | 4.63 [0.04; 6.42] | 6.02 [5.38; 6.60] |
|  | Female | 6.79 [1.30; 9.11] | 6.44 [3.69; 8.18] |
| Psychotic disorders | Male | 8.02 [2.64; 10.76] | 7.15 [6.56; 7.74] |
|  | Female | 8.73 [2.97; 11.88] | 6.77 [3.81; 8.78] |
| Bipolar disorder | Male | 1.31 [-6.24; 3.22] | 4.17 [3.14; 4.91] |
|  | Female | 3.37 [-1.93; 7.60] | 4.19 [1.57; 6.04] |
| Common mental disorders | Male | 3.28 [2.22; 4.24] | 3.93 [3.46; 4.37] |
|  | Female | 2.61 [0.87; 4.82] | 3.89 [2.13; 5.26] |
| Depressive disorders | Male | 4.19 [2.97; 5.32] | 4.18 [3.71; 4.66] |
|  | Female | 2.82 [0.98; 5.15] | 4.58 [2.63; 5.99] |
| Anxiety disorders | Male | 2.75 [0.84; 3.91] | 3.69 [3.18; 4.15] |
|  | Female | 1.83 [-0.09; 4.41] | 3.56 [1.63; 5.33] |
| Substance use disorders | Male | 3.62 [-3.16; 6.55] | 5.70 [5.02; 6.08] |
|  | Female | 9.59 [1.36; 14.03] | 7.16 [4.93; 9.02] |
| Any mental disorder except substance use disorders | Male | 3.40 [2.42; 4.27] | 4.08 [3.63; 4.53] |
|  | Female | 2.97 [1.34; 5.01] | 3.93 [2.19; 5.32] |

^a^ Data are stratified by region and sex. Negative LYL signify a longer lifespan among those with mental health diagnoses compared to those without.

**Supplemental Table 6:** All-cause life-years lost (LYL) associated with mental health diagnoses in the North America region between Jan 1, 2011 and Jan 26, 2021.

| **Diagnosis** | **Sex** | **Life-years lost^a^ [95% confidence intervals]** |
| --- | --- | --- |
| Any mental disorder | Male | 4.60 [4.01; 5.15] |
|  | Female | 4.82 [3.09; 6.60] |
| Severe mental disorders | Male | 4.62 [4.04; 5.17] |
|  | Female | 4.49 [2.67; 6.31] |
| Psychotic disorders | Male | 4.85 [4.24; 5.39] |
|  | Female | 5.18 [3.06; 7.02] |
| Bipolar disorder | Male | 4.72 [4.09; 5.27] |
|  | Female | 4.18 [2.51; 6.39] |
| Common mental disorders | Male | 6.71 [5.88; 7.35] |
|  | Female | 7.94 [4.37; 10.48] |
| Depressive disorders | Male | 7.65 [6.82; 8.36] |
|  | Female | 7.68 [4.14; 10.85] |
| Anxiety disorders | Male | 5.06 [3.94; 6.01] |
|  | Female | 5.80 [2.55; 7.89] |
| Substance use disorders | Male | 6.21 [5.32; 6.72] |
|  | Female | 6.90 [4.81; 8.96] |
| Any mental disorder except substance use disorders | Male | 4.73 [4.14; 5.25] |
|  | Female | 4.58 [2.87; 6.37] |

^a^ Data are stratified by region and sex. Negative LYL signify a longer lifespan among those with mental health diagnoses compared to those without.

**Supplemental Table 7:** All-cause life-years lost (LYL) associated with mental health diagnoses before the COVID-19 pandemic (March 1, 2020).

|  |  | **Life-years lost^a^ [95% confidence intervals]** | |
| --- | --- | --- | --- |
| **Diagnosis** | **Sex** | **South Africa** | **North America** |
| Any mental disorder | Male | 3.69 [2.58; 4.82] | 4.22 [3.76; 4.62] |
|  | Female | 3.60 [1.33; 5.81] | 4.69 [2.98; 6.02] |
| Severe mental disorders | Male | 5.83 [0.62; 8.47] | 6.07 [5.45; 6.67] |
|  | Female | 7.35 [0.76; 9.97] | 6.51 [3.64; 8.37] |
| Psychotic disorders | Male | 9.00 [2.56; 12.21] | 7.16 [6.57; 7.71] |
|  | Female | 10.00 [3.42; 13.18] | 6.76 [3.87; 8.90] |
| Bipolar disorder | Male | 1.78 [-6.28; 4.57] | 4.18 [3.23; 4.98] |
|  | Female | 2.75 [-2.72; 7.43] | 4.53 [1.61; 6.04] |
| Common mental disorders | Male | 3.60 [2.38; 4.82] | 3.97 [3.52; 4.38] |
|  | Female | 2.98 [0.76; 5.49] | 3.92 [2.17; 5.29] |
| Depressive disorders | Male | 3.92 [2.09; 5.44] | 4.21 [3.74; 4.65] |
|  | Female | 2.24 [0.09; 4.41] | 4.65 [2.54; 6.10] |
| Anxiety disorders | Male | 3.69 [1.31; 4.82] | 3.69 [3.22; 4.18] |
|  | Female | 2.88 [0.06; 5.54] | 3.60 [1.81; 5.41] |
| Substance use disorders | Male | 4.88 [-1.68; 7.98] | 5.70 [5.06; 6.08] |
|  | Female | 10.12 [0.02; 15.16] | 7.32 [5.35; 9.11] |
| Any mental disorder except substance use disorders | Male | 3.66 [2.55; 4.80] | 4.12 [3.67; 4.53] |
|  | Female | 3.62 [1.35; 5.82] | 3.99 [2.31; 5.33] |

^a^ Data are stratified by region and sex. Negative LYL signify a longer lifespan among those with mental health diagnoses compared to those without.

**Supplemental Table 8:** Life-years lost (LYL) associated with mental health diagnoses by cause of death in South Africa.

|  |  | **Life-years lost^a^ [95% confidence intervals]** | | | |
| --- | --- | --- | --- | --- | --- |
| **Diagnosis** | **Sex** | **All-causes** | **Natural causes** | **Unnatural causes** | **Unknown causes** |
| Any mental disorder | Male | 3.42 [2.44; 4.29] | 2.62 [1.58; 3.61] | 0.63 [0.22; 1.09] | 0.17 [-0.08; 0.49] |
|  | Female | 2.95 [1.30; 4.99] | 3.12 [1.52; 5.15] | 0.12 [-0.12; 0.33] | -0.29 [-0.59; -0.04] |
| Severe disorders | Male | 4.63 [0.04; 6.42] | 4.34 [-0.62; 6.26] | 0.54 [-0.20; 1.43] | -0.25 [-0.40; -0.12] |
|  | Female | 6.79 [1.30; 9.11] | 6.83 [1.35; 9.42] | 0.25 [-0.20; 0.77] | -0.29 [-0.62; 0.00] |
| Psychotic disorders | Male | 8.02 [2.64; 10.76] | 7.11 [1.39; 10.28] | 1.16 [-0.51; 2.89] | -0.25 [-0.41; -0.12] |
|  | Female | 8.73 [2.97; 11.88] | 8.50 [3.09; 11.56] | 0.58 [-0.35; 1.79] | -0.35 [-0.65; -0.12] |
| Bipolar disorder | Male | 1.31 [-6.24; 3.22] | 1.28 [-6.09; 3.38] | 0.27 [-0.68; 1.37] | -0.25 [-0.40; -0.12] |
|  | Female | 3.37 [-1.93; 7.60] | 3.41 [-1.70; 7.65] | 0.24 [-0.29; 0.85] | -0.28 [-0.62; 0.03] |
| Common disorders | Male | 3.28 [2.22; 4.24] | 2.58 [1.42; 3.58] | 0.68 [0.26; 1.17] | 0.03 [-0.18; 0.30] |
|  | Female | 2.61 [0.87; 4.82] | 2.76 [1.13; 4.87] | 0.14 [-0.10; 0.37] | -0.29 [-0.59; -0.04] |
| Depressive disorders | Male | 4.19 [2.97; 5.32] | 3.27 [1.98; 4.55] | 0.82 [0.26; 1.68] | 0.09 [-0.16; 0.44] |
|  | Female | 2.82 [0.98; 5.15] | 2.98 [1.15; 5.27] | 0.15 [-0.09; 0.40] | -0.30 [-0.61; -0.05] |
| Anxiety disorders | Male | 2.75 [0.84; 3.91] | 2.40 [0.49; 3.60] | 0.43 [0.06; 0.86] | -0.08 [-0.27; 0.12] |
|  | Female | 1.83 [-0.09; 4.41] | 1.98 [0.09; 4.49] | 0.15 [-0.12; 0.38] | -0.30 [-0.61; -0.06] |
| Substance use disorders | Male | 3.62 [-3.16; 6.55] | 2.64 [-4.22; 5.46] | 1.22 [0.06; 2.50] | -0.24 [-0.39; -0.13] |
|  | Female | 9.59 [1.36; 14.03] | 8.78 [0.53; 13.14] | 1.19 [-0.15; 2.79] | -0.39 [-0.73; -0.13] |
| Any mental disorder except substance use disorders | Male | 3.40 [2.42; 4.27] | 2.59 [1.54; 3.58] | 0.64 [0.23; 1.10] | 0.17 [-0.08; 0.50] |
|  | Female | 2.97 [1.34; 5.01] | 3.14 [1.55; 5.17] | 0.12 [-0.12; 0.33] | -0.29 [-0.59; -0.04] |

^a^ Data are stratified by sex. Negative LYL signify a longer lifespan among those with mental health diagnoses compared to those without.

**Supplemental Table 9:** Life-years lost (LYL) associated with mental health diagnoses by cause of death and sex in North America.

|  |  | **Life-years lost^a^ [95% confidence intervals]** | | | | | |
| --- | --- | --- | --- | --- | --- | --- | --- |
| **Diagnosis** | **Sex** | **All-causes** | **Natural causes** | **Accident/injury** | **Homicides** | **Suicides** | **Unknown causes** |
| Any mental disorder | Male | 4.28 [3.75; 4.68] | 3.11 [2.58; 3.52] | 0.61 [0.46; 0.76] | 0.05 [0.00; 0.09] | 0.09 [0.00; 0.17] | 0.43 [0.24; 0.61] |
|  | Female | 4.35 [1.01; 6.70] | 4.96 [2.63; 6.91] | 0.78 [-0.03; 1.42] | -0.14 [-0.35; 0.02] | 0.04 [0.00; 0.10] | -1.29 [-3.30; 0.11] |
| Severe mental disorders | Male | 6.41 [5.67; 6.90] | 3.97 [3.20; 4.52] | 1.09 [0.80; 1.36] | 0.05 [-0.02; 0.12] | 0.25 [0.14; 0.36] | 1.04 [0.72; 1.37] |
|  | Female | 8.14 [2.04; 10.52] | 7.25 [3.02; 8.87] | 1.93 [0.38; 2.97] | 0.00 [-0.19; 0.20] | -0.01 [-0.05; 0.00] | -1.02 [-4.39; 1.27] |
| Psychotic disorders | Male | 7.26 [6.68; 7.97] | 4.76 [4.17; 5.52] | 1.03 [0.72; 1.32] | 0.04 [-0.03; 0.12] | 0.18 [0.07; 0.29] | 1.25 [0.88; 1.60] |
|  | Female | 8.31 [2.14; 11.59] | 7.50 [3.58; 9.87] | 1.86 [0.33; 2.95] | -0.09 [-0.21; -0.01] | -0.02 [-0.06; 0.00] | -0.94 [-5.13; 1.98] |
| Bipolar disorder | Male | 4.37 [3.16; 5.13] | 1.89 [0.65; 2.65] | 1.40 [1.02; 1.80] | 0.05 [-0.04; 0.14] | 0.39 [0.22; 0.58] | 0.65 [0.23; 1.07] |
|  | Female | 6.83 [0.27; 9.15] | 6.38 [0.99; 8.16] | 1.41 [0.05; 2.75] | 0.04 [-0.20; 0.32] | 0.00 [-0.02; 0.00] | -0.99 [-4.16; 1.14] |
| Common mental disorders | Male | 3.87 [3.36; 4.28] | 2.76 [2.23; 3.20] | 0.63 [0.48; 0.80] | 0.04 [-0.01; 0.08] | 0.11 [0.02; 0.19] | 0.33 [0.14; 0.52] |
|  | Female | 4.11 [0.74; 6.59] | 4.76 [2.38; 6.69] | 0.77 [-0.14; 1.46] | -0.13 [-0.33; 0.02] | 0.02 [0.00; 0.07] | -1.32 [-3.40; 0.16] |
| Depressive disorders | Male | 4.03 [3.53; 4.45] | 2.82 [2.26; 3.29] | 0.69 [0.52; 0.86] | 0.05 [0.00; 0.10] | 0.13 [0.04; 0.21] | 0.33 [0.12; 0.55] |
|  | Female | 4.54 [1.04; 7.04] | 5.15 [2.58; 7.12] | 0.80 [-0.19; 1.49] | -0.12 [-0.32; 0.03] | 0.02 [0.00; 0.08] | -1.32 [-3.59; 0.21] |
| Anxiety disorders | Male | 3.76 [3.26; 4.24] | 2.45 [1.92; 2.94] | 0.73 [0.57; 0.90] | 0.04 [0.00; 0.09] | 0.11 [0.03; 0.19] | 0.42 [0.19; 0.64] |
|  | Female | 3.86 [0.32; 6.74] | 4.35 [2.03; 6.54] | 0.84 [0.05; 1.41] | -0.09 [-0.23; 0.03] | 0.00 [-0.02; 0.00] | -1.24 [-4.15; 0.68] |
| Substance use disorders | Male | 6.07 [5.33; 6.44] | 4.14 [3.38; 4.54] | 1.00 [0.83; 1.21] | 0.07 [0.01; 0.12] | 0.13 [0.04; 0.21] | 0.73 [0.48; 0.98] |
|  | Female | 7.29 [3.81; 9.73] | 7.37 [5.09; 9.66] | 1.31 [0.16; 1.95] | -0.10 [-0.33; 0.08] | 0.04 [0.00; 0.13] | -1.32 [-3.73; 0.40] |
| Any mental disorder except substance use disorders | Male | 4.07 [3.57; 4.48] | 2.90 [2.37; 3.35] | 0.62 [0.47; 0.79] | 0.04 [-0.01; 0.08] | 0.11 [0.03; 0.19] | 0.40 [0.20; 0.59] |
|  | Female | 4.33 [0.99; 6.62] | 4.85 [2.45; 6.83] | 0.84 [-0.04; 1.50] | -0.13 [-0.33; 0.02] | 0.02 [0.00; 0.07] | -1.26 [-3.30; 0.20] |

^a^ The study population is restricted to cohorts and time periods with available cause of death data. Negative LYL signify a longer lifespan among those with mental health diagnoses compared to those without.

**Supplemental Table 10**: CD4 cell count at the time of a death from natural causes, by region, sex, and mental health status.

|  | **Male** | |  | **Female** | |
| --- | --- | --- | --- | --- | --- |
|  | Received mental health diagnoses | Without mental health diagnoses |  | Received mental health diagnoses | Without mental health diagnoses |
| **South Africa** | N=1590 | N=2502 |  | N=1228 | N=1563 |
| CD4, cells/µL |  |  |  |  |  |
| <200 | 750 (47.2%) | 1371 (54.8%) |  | 569 (46.3%) | 803 (51.4%) |
| 200-349 | 242 (15.2%) | 350 (14.0%) |  | 167 (13.6%) | 195 (12.5%) |
| ≥350 | 439 (27.6%) | 476 (19.0%) |  | 364 (29.6%) | 348 (22.3%) |
| Unknown^a^ | 159 (10.0%) | 305 (12.2%) |  | 128 (10.4%) | 217 (13.9%) |
| Median [IQR] | 185 [51, 414] | 124 [37, 312] |  | 190 [54, 479] | 132 [36, 355] |
| Standardized difference^b^ | 0.202 |  |  | 0.181 |  |
| **North America** | N=9248 | N=2778 |  | N=713 | N=205 |
| CD4, cells/µL |  |  |  |  |  |
| <200 | 4375 (47.3%) | 1398 (50.3%) |  | 361 (50.6%) | 112 (54.6%) |
| 200-349 | 1663 (18.0%) | 440 (15.8%) |  | 108 (15.1%) | 30 (14.6%) |
| ≥350 | 2462 (26.6%) | 640 (23.0%) |  | 188 (26.4%) | 37 (18.0%) |
| Unknown^a^ | 748 (8.1%) | 300 (10.8%) |  | 56 (7.9%) | 26 (12.7%) |
| Median [IQR] | 191 [58, 386] | 160 [37, 358] |  | 165 [39, 402] | 121 [21, 294] |
| Standardized difference^b^ | 0.082 |  |  | 0.267 |  |

^a^ No CD4 cell count measurement available within one year of the natural death. ^c^ Comparing CD4 at natural death between people who received a mental health diagnosis and people who didn’t.

**Supplemental Table 11:** Life-years lost (LYL) due to natural causes associated with mental health diagnoses in South Africa, by sex and by CD4 cell count at death.

|  |  | **Life-years lost^a^ [95% confidence intervals]** | | | | |
| --- | --- | --- | --- | --- | --- | --- |
| **Diagnosis** | **Sex** | **Natural causes (total)** | **CD4<200 cells/µL** | **CD4 200-349 cells/µL** | **CD4≥350 cells/µL** | **CD4 unknown^b^** |
| Any mental disorder | Male | 2.62 [1.58; 3.61] | 0.78 [-0.19; 1.80] | 0.10 [-0.55; 0.81] | 1.96 [0.93; 2.97] | -0.22 [-0.91; 0.57] |
|  | Female | 3.12 [1.52; 5.15] | 1.00 [0.04; 2.17] | 0.19 [-0.57; 1.12] | 1.97 [0.55; 3.41] | -0.04 [-0.74; 0.68] |
| Severe mental disorders | Male | 4.34 [-0.62; 6.26] | 0.44 [-1.68; 2.18] | 1.76 [-0.19; 5.69] | 0.07 [-1.30; 1.96] | 2.06 [-2.09; 3.66] |
|  | Female | 6.83 [1.35; 9.42] | 2.47 [-0.09; 5.04] | 0.62 [-0.78; 2.51] | 4.94 [-0.11; 6.88] | -1.21 [-1.73; -0.70] |
| Psychotic disorders | Male | 7.11 [1.39; 10.28] | 1.53 [-1.51; 4.00] | 3.02 [-0.43; 6.74] | 0.84 [-1.16; 3.19] | 1.72 [-1.66; 3.49] |
|  | Female | 8.50 [3.09; 11.56] | 2.44 [-0.82; 5.83] | 1.81 [-0.64; 4.68] | 4.83 [0.24; 7.46] | -0.58 [-1.59; 0.67] |
| Bipolar disorder | Male | 1.28 [-6.09; 3.38] | -1.22 [-3.41; 0.68] | 4.72 [-0.95; 6.36] | -0.10 [-2.01; 2.32] | -2.12 [-2.63; -1.55] |
|  | Female | 3.41 [-1.70; 7.65] | 3.04 [-0.56; 6.40] | -0.03 [-1.50; 1.93] | 1.96 [-1.00; 5.12] | -1.55 [-2.05; -1.14] |
| Common mental disorders | Male | 2.58 [1.42; 3.58] | 0.91 [-0.13; 1.95] | -0.06 [-0.74; 0.68] | 1.89 [0.80; 2.95] | -0.18 [-0.93; 0.67] |
|  | Female | 2.76 [1.13; 4.87] | 1.04 [0.07; 2.26] | 0.20 [-0.58; 1.12] | 1.53 [0.15; 3.03] | -0.02 [-0.79; 0.80] |
| Depressive disorders | Male | 3.27 [1.98; 4.55] | 1.26 [0.12; 2.68] | 0.03 [-0.75; 0.87] | 1.95 [0.74; 3.13] | 0.03 [-0.84; 1.09] |
|  | Female | 2.98 [1.15; 5.27] | 1.20 [0.23; 2.34] | 0.15 [-0.74; 1.22] | 1.96 [0.26; 3.79] | -0.34 [-0.90; 0.18] |
| Anxiety disorders | Male | 2.40 [0.49; 3.60] | 0.81 [-0.60; 2.36] | 0.41 [-0.87; 2.01] | 1.72 [0.31; 3.60] | -0.53 [-1.23; 0.07] |
|  | Female | 1.98 [0.09; 4.49] | 0.56 [-0.42; 1.82] | 0.21 [-0.66; 1.34] | 1.10 [-0.37; 2.98] | 0.10 [-0.84; 1.41] |
| Substance use disorders | Male | 2.64 [-4.22; 5.46] | -0.61 [-3.10; 2.47] | 4.12 [-1.03; 6.67] | -1.04 [-2.56; 0.65] | 0.17 [-1.43; 1.65] |
|  | Female | 8.78 [0.53; 13.14] | 2.64 [-1.79; 8.67] | -0.52 [-2.16; 1.92] | 7.91 [-0.51; 14.44] | -1.25 [-2.12; 0.00] |
| Any mental disorder except substance use disorders | Male | 2.59 [1.54; 3.58] | 0.78 [-0.19; 1.79] | 0.06 [-0.60; 0.78] | 1.97 [0.94; 2.97] | -0.23 [-0.93; 0.57] |
|  | Female | 3.14 [1.55; 5.17] | 1.00 [0.04; 2.18] | 0.20 [-0.56; 1.13] | 1.97 [0.55; 3.41] | -0.04 [-0.74; 0.68] |

^a^ Negative LYL signify a longer lifespan among those with mental health diagnoses compared to those without. ^b^ No CD4 available within one year of the individual’s death

**Supplemental Table 12:** Life-years lost (LYL) due to natural causes associated with mental health diagnoses in North America, by sex and by CD4 cell count at death.

|  |  | **Life-years lost^a^ [95% confidence intervals]** | | | | |
| --- | --- | --- | --- | --- | --- | --- |
| **Diagnosis** | **Sex** | **Natural causes (total)** | **CD4<200 cells/µL** | **CD4 200-349 cells/µL** | **CD4≥350 cells/µL** | **CD4 unknown^b^** |
| Any mental disorder | Male | 3.11 [2.58; 3.52] | 0.96 [0.57; 1.35] | 0.86 [0.57; 1.11] | 1.36 [0.98; 1.68] | -0.07 [-0.24; 0.10] |
|  | Female | 4.96 [2.63; 6.91] | 1.77 [0.25; 3.08] | 1.12 [0.08; 1.81] | 1.94 [0.47; 3.28] | 0.12 [-0.44; 0.70] |
| Severe mental disorders | Male | 3.97 [3.20; 4.52] | 1.13 [0.62; 1.71] | 0.85 [0.47; 1.23] | 2.06 [1.42; 2.45] | -0.07 [-0.31; 0.15] |
|  | Female | 7.25 [3.02; 8.87] | 1.86 [-0.46; 3.67] | 1.76 [-0.08; 3.22] | 3.35 [0.40; 5.13] | 0.28 [-0.49; 1.17] |
| Psychotic disorders | Male | 4.76 [4.17; 5.52] | 2.05 [1.52; 2.72] | 0.92 [0.54; 1.36] | 1.80 [1.35; 2.30] | -0.01 [-0.24; 0.24] |
|  | Female | 7.50 [3.58; 9.87] | 3.15 [0.66; 5.31] | 1.20 [-0.28; 2.91] | 2.92 [-0.06; 4.82] | 0.23 [-0.61; 1.30] |
| Bipolar disorder | Male | 1.89 [0.65; 2.65] | -0.27 [-0.89; 0.34] | 0.46 [0.00; 0.96] | 1.77 [0.84; 2.33] | -0.07 [-0.44; 0.36] |
|  | Female | 6.38 [0.99; 8.16] | 0.55 [-2.09; 2.46] | 2.37 [-0.57; 3.73] | 3.48 [0.62; 5.39] | -0.03 [-0.78; 0.71] |
| Common mental disorders | Male | 2.76 [2.23; 3.20] | 0.76 [0.39; 1.15] | 0.72 [0.43; 1.00] | 1.39 [1.00; 1.75] | -0.11 [-0.28; 0.06] |
|  | Female | 4.76 [2.38; 6.69] | 1.58 [0.05; 2.86] | 1.16 [0.16; 1.80] | 1.86 [0.24; 3.37] | 0.17 [-0.43; 0.83] |
| Depressive disorders | Male | 2.82 [2.26; 3.29] | 0.79 [0.43; 1.17] | 0.71 [0.41; 0.97] | 1.43 [1.05; 1.81] | -0.11 [-0.29; 0.04] |
|  | Female | 5.15 [2.58; 7.12] | 1.76 [0.27; 3.13] | 1.10 [0.18; 1.88] | 1.93 [0.22; 3.48] | 0.36 [-0.38; 1.09] |
| Anxiety disorders | Male | 2.45 [1.92; 2.94] | 0.43 [-0.02; 0.77] | 0.71 [0.43; 1.02] | 1.48 [1.13; 1.87] | -0.16 [-0.35; 0.01] |
|  | Female | 4.35 [2.03; 6.54] | 0.90 [-0.58; 2.47] | 1.11 [0.35; 1.89] | 2.12 [0.09; 3.76] | 0.22 [-0.43; 0.82] |
| Substance use disorders | Male | 4.14 [3.38; 4.54] | 1.39 [0.93; 1.86] | 1.01 [0.69; 1.33] | 1.78 [1.14; 2.08] | -0.03 [-0.22; 0.14] |
|  | Female | 7.37 [5.09; 9.66] | 2.41 [0.94; 3.87] | 1.34 [0.37; 2.29] | 3.57 [1.65; 5.21] | 0.04 [-0.49; 0.54] |
| Any mental disorder except substance use disorders | Male | 2.90 [2.37; 3.35] | 0.83 [0.47; 1.22] | 0.79 [0.51; 1.07] | 1.36 [0.97; 1.71] | -0.09 [-0.26; 0.07] |
|  | Female | 4.85 [2.45; 6.83] | 1.71 [0.28; 3.03] | 1.13 [0.08; 1.78] | 1.86 [0.27; 3.30] | 0.15 [-0.45; 0.78] |

^a^ Negative LYL signify a longer lifespan among those with mental health diagnoses compared to those without. ^b^ No CD4 available within one year of the individual’s death

**Supplemental Figure 1**: Psychiatric comorbidity among participants from South Africa and North America.

The figure shows the percentages of individuals with a given mental health diagnosis (y-axis) who received another mental health diagnosis (x-axis) during their follow-up time. Darker colors represent higher values.


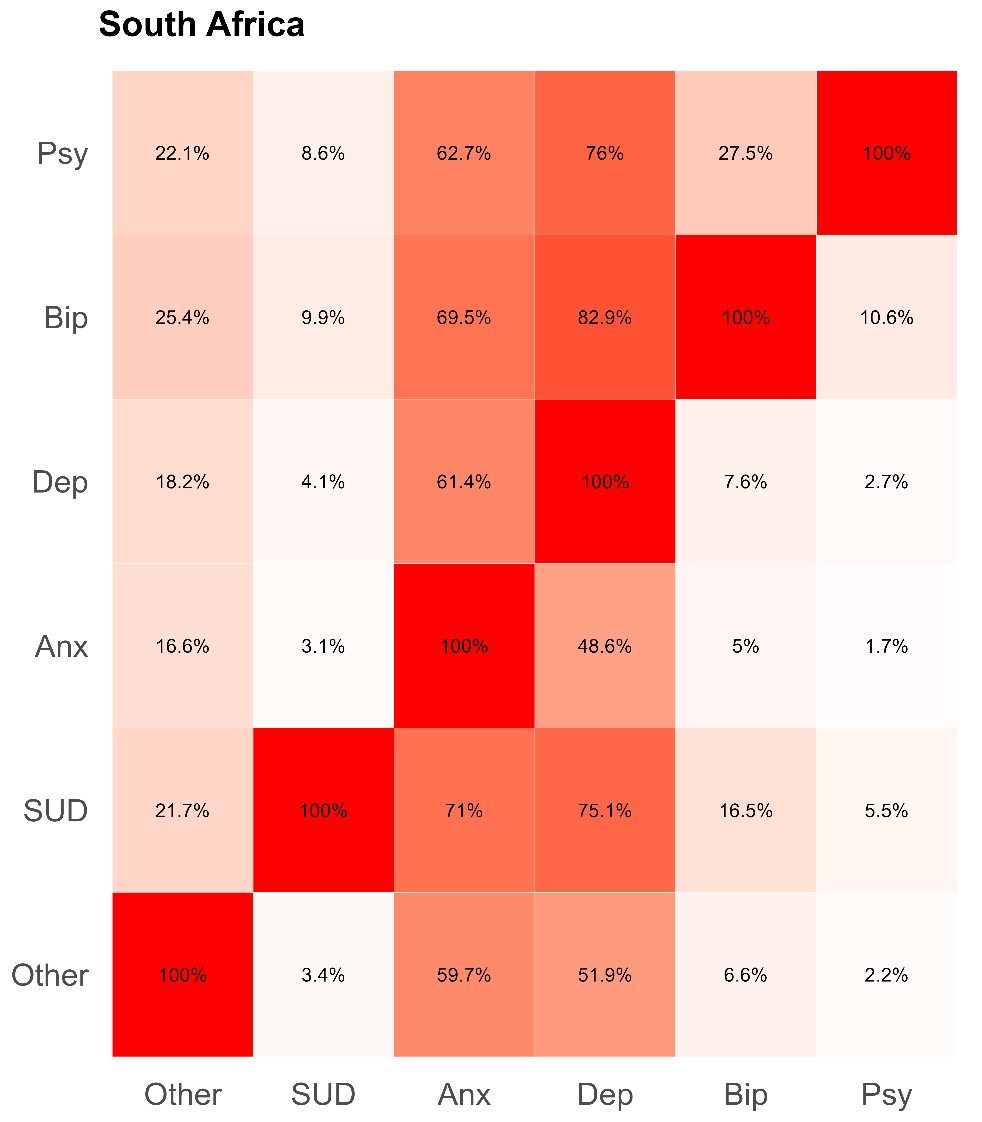

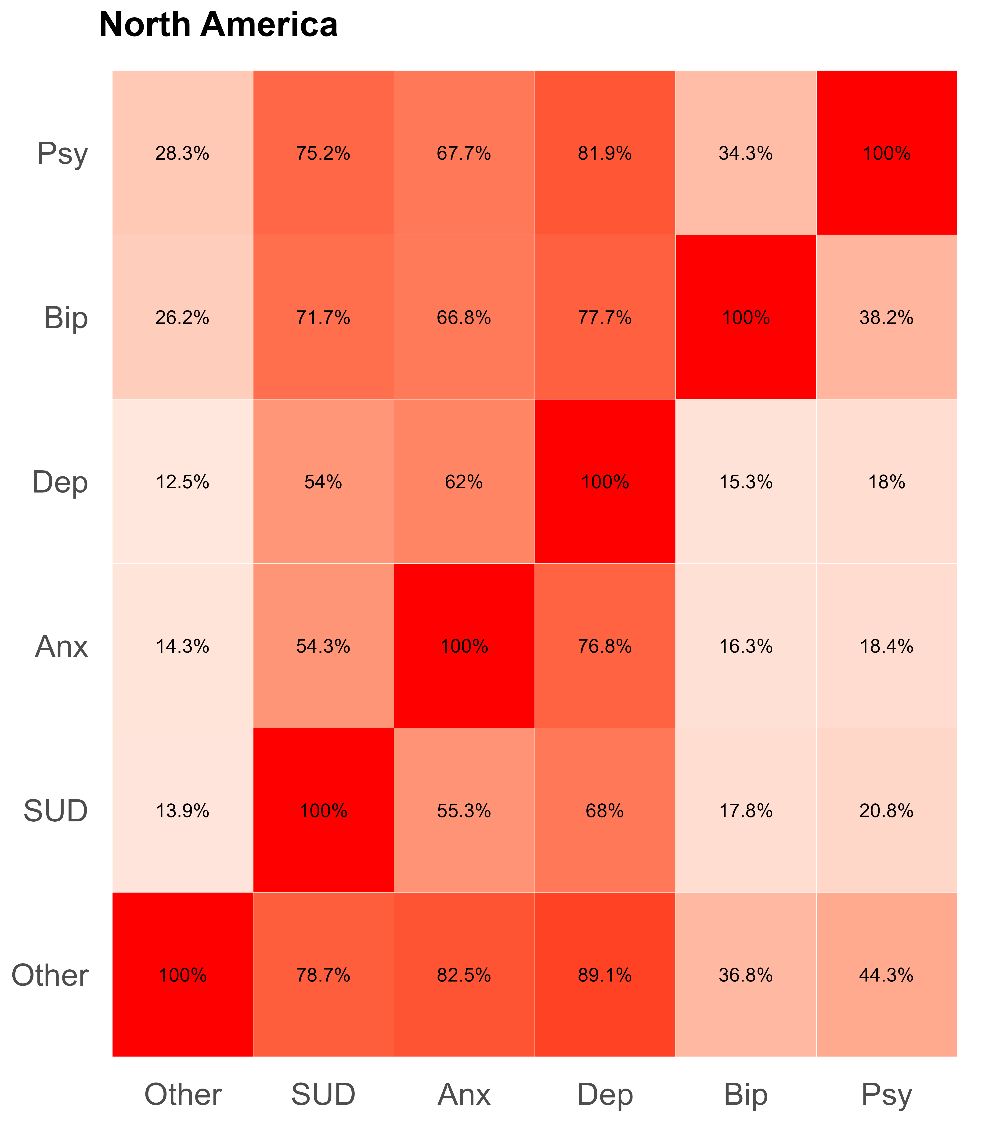


SUD=substance use disorder, Psy=psychotic disorder, Bip=bipolar disorder, Dep=depressive disorder, Anx=anxiety disorder, Other=other mental disorder
